# Extraversion personality, perceived health and activity participation among community-dwelling aging adults in Hong Kong

**DOI:** 10.1371/journal.pone.0209154

**Published:** 2018-12-12

**Authors:** Daniel W. L. Lai, Nan Qin

**Affiliations:** 1 Department of Applied Social Sciences, The Hong Kong Polytechnic University, Hung Hom, Kowloon, Hong Kong; 2 School of Humanities and Communication, Guangdong University of Finance and Economics, Guangzhou, China; University of Florence, ITALY

## Abstract

Activity participation is essential to the wellbeing of aging adults. Divergent levels of activity participation within aging populations have been explained from diverse perspectives, but the interaction effects of key determinants, such as personality and health, are often ignored. This study examines the effects of extravert personality on aging adults’ activity levels by addressing its interaction with perceived physical health and mental health. A sample of 304 adults aged 50 and older was selected using systematic sampling from participants of an institute for promoting active aging at a university in Hong Kong in 2017. Data on socio-demographic characteristics, perceived physical and mental health, extraversion personality traits, and level of activity participation were collected using a telephone survey. Most participants (46.7%) reported moderate activity levels and over a quarter (26.6%) reported high or low activity levels. Multi-nominal logistic regression analyses show that extraversion was associated with an increased likelihood of reporting moderate (OR = 1.85, p = .036) but not high (p > .05) activity levels when adjusted for perceived physical and mental health and socio-demographics, with low activity levels being the constant comparison. Meanwhile, extraversion predicted both moderate (OR = 3.84, p = .014) and high (OR = 5.06, p = .032) activity levels for participants with poor or average perceived mental health. However, the interaction effects of extraversion with perceived physical health or mental health were not significant in predicting either moderate or high activity levels (p > .05). The implications for enhancing activity participation among aging adults are discussed in view of both personality and perceived health status.

## Introduction

Activity participation is a major determinant of quality of life as people age [1]. As a key component of active aging, activity participation influences aging adults’ wellbeing in many aspects [2]. Existing literature has documented the positive effects of participation in physical, cognitive, social, leisure, and civic activities on physical health, mortality and disease, mental health, cognitive capacity, functional maintenance, life satisfaction, and social support [3–8].

While the benefits of activity participation are well recognized in existing research, it is vital to understand which factors encourage and enable aging adults to engage in activities. A significant body of literature has revealed individual characteristics associated with participation in activities (e.g. productive, leisure, social activities) among aging adults. In addition to socio-demographic factors (e.g. being female, younger age, white race, more income, and higher education) [2, 9, 10], physical and mental health (better status) [2, 10–12] and personality traits such as higher extraversion or lower neuroticism [13, 14] have been identified as the most significant influencing factors. Interpersonal factors (e.g. living with others, having larger social networks) [10, 15] and environmental characteristics (e.g. urban context, transportation availability) [16, 17] are also determinants. Different theoretical perspectives have explained activity participation among aging adults. For example, the theory of planned behavior [18–20] elucidates that actual behaviors related to activity participation can be ascribed to the interaction between an intention (or motivation) and perceived control (e.g. ability, resources, opportunities) to participate in activities. The model of constraints [21] further elaborates influential factors as barriers to activity choices and participation behaviors. Health status, psychological attributes, perceived social pressure, socialized values and beliefs, perception of one’s ability, and opportunity to participate in an activity represent “intrapersonal barriers”. Influence or interference by other people on one’s activity preference or participation and lack of a partner in group activities represent “interpersonal barriers”. Constraints resulting from financial resources, information, environmental conditions, and competing roles (e.g. familial tasks, work) represent “structural barriers”. An empirical study tested the integration of the theory of planned behavior and the model of constraints and found that intrapersonal factors (health-related problems, lack of confidence, feeling tired, worry about pain) were the most significant barriers and mitigated people’s intention for activity participation via attitudes towards the activity behavior and perceived behavioral control [22]. The aforementioned theoretical models provide helpful perspectives for understanding the relationship between internal and external forces that drive activity participation. However, these perspectives have not considered the relationship between specific intrapersonal factors such as personality and health, and their respective effects. This study will focus on the influence of personality on activity participation by considering its interaction with health status.

### Personality and activity participation

Previous research has demonstrated that extraversion is one of the most important personality traits shaping activity participation [23, 24]. Extraversion refers to “an energetic approach toward the social and material worlds and includes traits such as sociability, activity, assertiveness and positive emotionality” [25], which implies explicit influence on activity participation. According to previous studies, extraversion is positively correlated with participation in physical, cognitive, and social activities among aging populations [7, 26]. Meta-analyses have also confirmed the significant association between extraversion and physical activity [14, 24]. Some personality features relevant to extraversion have been found to affect activity participation in aging adults. For instance, feelings of shyness and awkwardness about physical activity limit participation [27], while self-esteem and optimism are related to increased social activity participation [28]. A recent study shows that extraversion predicts increased participation in a variety of activities across the lifespan, independent of disease burden [13]. However, Furnham [29] argued that the association between extraversion and activity participation could be affected by various factors, including health status. It is essential to clarify the association between extraversion and activity participation in aging adults when taking health conditions into account.

### Physical health and activity participation

While accumulated evidence indicates that activity participation benefits the health of aging adults, physical impairment can represent a barrier to participation [30–32]. The World Health Organization [33] has reported that health conditions and functional impairments contribute to activity limitations and restrict participation in learning, communication, mobility, social interaction, and civic life. Empirical studies lend support to the barrier effect. For instance, physical frailty in strength, balance, and flexibility have been found to limit physical activity [27] while disabilities, health conditions (e.g. arthritis, incontinence), and functional health problems (e.g. hearing difficulties) limit social engagement and civic activities (e.g. volunteering) [7, 32, 34–38]. Individual characteristics such as age might influence health effects on activity participation. For example, a population-based study showed that cancer is significantly more likely to reduce participation in survivors aged 60 or older than in younger survivors [39]. However, these studies have not clearly examined differences in relationships between physical health impairment and activity limitation among aging people with divergent demographic or personality traits.

### Mental health and activity participation

Research findings concerning the influence of mental health on activity participation among aging adults are inconclusive. A number of studies confirm the model of constraints, as mental health and cognitive challenges might limit individuals’ activity participation. For example, depression has been found to be inversely correlated with participation in cognitive activities [7]. Cognitive impairment has been associated with a decline in participation in physical, passive, cultural, and leisure activities [40], while social cognition has been positively associated with social functioning [41]. In addition to direct impacts on self-perceived control (capacity) and motivation, mental impairment might also affect interpersonal barriers to activity participation, such as rejection and exclusion by others. Taylor and Field [42] identified connections between stigmatization and functional limitation, self-esteem, and withdrawal from social activities. However, there is also empirical evidence for an inverse compensation effect of poor mental health on activity participation. For example, depression has been positively related to formal volunteering activities, as aging people experiencing depression may strive for social engagement to increase social support and sense of belonging [32]. It is unclear which factors influence the contradictory relationships between mental health and activity participation, although previous studies suggest that psychological functioning or personality traits might explain these relationships [32, 42]. This illustrates the need for additional research on the associations between mental health, activity participation, and mediating factors.

Existing literature has demonstrated that extravert personality plays an important role in determining activity participation in aging adults, but *how* this relationship is influenced by physical and mental health has not been explored. The potential effects of interactions between these variables on aging adults’ intention and capacity for activity participation also require further examination. Furthermore, previous studies on activity participation focused on specific types of activities, such as daily routines or civic or religious activity [1, 2, 6, 28, 43, 44]. However, aging people are often engaged in diverse types of activities that are rarely fully captured, and some researchers argue for the importance and advantage of creating an aggregate measure for gauging activity levels [13, 45].This study addressed two main research questions: 1) To what extent does an extravert personality predict the likelihood and level of participation in activities among aging adults? 2) To what extent do extraversion and perceived physical and mental health interact in predicting activity level? Based on previous literature reviewed above, it is hypothesized that: 1) Highly extravert aging adults will be more likely to have a higher activity level, and 2) perceived physical and mental health will moderate the relationship between extraversion and activity level.

## Methodology

### Research design and data collection

This study employed a quantitative approach, with data collected from adults aged 50 or older in Hong Kong via telephone survey. The survey sample was recruited from registrants of non-interventional programs (including interest classes, talks, etc.) run by an institute of active aging affiliated with a university at Hong Kong, which issued ethical approval for this study. A total of 1,729 registrants were ranked based on their participation hours (ranging from 1 to 263 hours with a mean of 15.63 and a standard deviation of 30.64) in the institute’s programs between October 2015 and September 2016. A systematic sampling method was used to select every other member on this list to be potential survey participants. A total of 869 cases were selected for the survey. A maximum of three calls were made for each contact number, resulting in the identification of 604 valid numbers. Of these, 304 cases consented to participate in the survey and completed the interview, resulting in a response rate of 50.3% (successful cases / valid phone numbers). Trained research assistants conducted telephone interviews in January 2017. Each interview lasted for approximately 30 minutes. A comparison of basic age, gender, and education backgrounds of the successful and refused cases in the administrative membership record system showed no significant difference in their demographic pattern.

### Measures

This study measured four main variables, including socio-demographics, perceived physical and mental health, extraversion, and level of activity participation.

#### Socio-demographics

Seven demographic variables were included. Gender was coded as *male* (0) or *female* (1). Age was a continuous variable coded in years. Education was an ordinal variable with three categories: *primary or below* (1), *secondary education* (2), and *college or above* (3). Financial status was coded in five categories ranging from *very poor* (1) to *very good* (5). Having children or not, living alone or not, and being retired or not were all dummy variables with positive responses coded as 1 and negative responses coded as 0.

#### Perceived physical and mental health

Participants rated their perceived physical health and mental health on a five-point ordinal measure ranging from *very poor* (1) to *very good* (5). Higher scores represent better perceived physical or mental health. Single-item measures have been recognized for their efficiency, cheapness, and face validity and have been widely adopted for self-rated health studies [2, 46].

#### Extraversion

The extraversion subscale of the Big Five Inventory [47, 48] was employed to measure the extravert traits of participants’ personality. The Big Five Inventory, an established instrument on personality, has been used in research with diverse populations. The Chinese version has been tested among aging Chinese adults in Hong Kong and demonstrated an acceptable internal consistency with an alpha of .68 [49]. Participants indicated their agreement on eight statements on a five-point scale. The total score was the average of all item responses, with higher scores representing greater extraversion. In this study, the scale had good internal consistency (Cronbach’s alpha = .76).

#### Level of activity participation

Participants were asked to respond to the question, “How was your activity level in participating in various types of activities in the previous year: low, moderate, or high?” Responses were coded as *low* = 1, *moderate* = 2, and *high* = 3.

### Data analysis

SPSS 20.0 was used to conduct data analysis. The associations between activity level and each variable were examined by comparing groups with low, moderate, and high activity levels using bivariate analyses, including crosstab for categorical variables (gender, education, having children or not, living alone or not, working status, financial status, perceived physical and mental health) and one-way ANOVA for continuous variables (age and extraversion). Multi-nominal logistic regression was employed to determine the odds ratio of higher levels of activity with socio-demographics, extravert personality, perceived physical health, perceived mental health, and interaction variables as predictors. Low activity level was used as the reference category. For model parsimony, perceived physical health and mental health were recoded into two categories (1 = poor or average, 2 = good or very good, with no reports of being very poor). Four different models were tested to hierarchically ascertain the independent effect of extravert personality and its interaction effects with perceived physical and mental health on activity level. Model 1 included socio-demographic and extraversion as predictors. Model 2 added perceived physical health and mental health. Their interaction effects with extraversion were entered into Model 3 furthermore, which were generated by multiplying perceived physical or mental health with extraversion using the function of “Custom/Stepwise”. Model 4 separated the data by perceived physical (or mental) health and tested the effect of extraversion and perceived mental (or physical) health independent of socio-demographics.

## Results

### Participants’ profile

The final sample (N = 304) consisted of 92 males (30.3%) and 212 females (69.7%), aged 50 to 86 years (mean = 63.46, SD = 6.85). More than half the participants (n = 156, 51.3%) had attained college-level education or above, 41.8% (n = 127) had attained secondary-level education, and 6.9% (n = 21) had completed primary education or below. The majority had one or more children (n = 239, 78.6%) and were living with others (n = 259, 85.2%). About 40% of participants reported good (n = 87, 28.6%) or very good (n = 28, 9.2%) financial status, 60.2% (n = 183) reported average financial status, and a small proportion (n = 6, 2.0%) were poorly off. Most were retired (n = 227, 74.7%). Participants perceived better mental health than physical health, with 64.4% (n = 196) perceiving good or very good mental health and 49.4% (n = 150) perceiving good or very good physical health. Concerning activity levels, 46.7% (n = 142) reported moderate levels, and low or high activity levels were each reported by 26.6% (n = 81). Table 1 presents participants’ profile by activity level. Chi-square tests indicated that participants living alone (χ^2^ = 10.92, p = .004) and perceiving good physical health (χ^2^ = 15.39, p < .001) and mental health (χ^2^ = 14.32, p < .001) were more likely to have a high activity level. Extraversion was positively associated with activity level (F = 6.00, p = .003). Participants with moderate and high activity levels were more likely to be extraverted than those with low activity levels, but there was no significant difference in extraversion between those with moderate and high activity levels.

**Table 1:** Participants’ profile by activity level (N = 304).

	Total (304, 100%)		Low activity (81, 26.6%)		Moderate activity (142, 46.7%)		High activity (81, 26.6%)		Chi-square test	
**Gender**	n	%	n	%	n	%	n	%	χ^2^	p
Male	92	100	22	23.9	42	45.7	28	30.4	1.12	.573
Female	212	100	59	27.8	100	47.2	53	25.0		
**Education**	n	%	n	%	n	%	n	%	χ^2^	p
Primary or below	21	100	8	38.1	8	38.1	5	23.8	2.77	.596
Secondary	127	100	34	26.8	63	49.6	30	23.6		
College or above	156	100	39	25.0	71	45.5	46	29.5		
**Have children**	n	%	n	%	n	%	n	%	χ^2^	p
No	65	100	22	33.8	26	40.0	17	26.2	2.41	.299
Yes	239	100	59	24.7	116	48.5	64	26.8		
**Living alone**	n	%	n	%	n	%	n	%		
No	259	100	68	26.3	130	50.2	61	23.6	10.92	.004
Yes	45	100	13	28.9	12	26.7	20	44.4		
**Financial status**	n	%	n	%	n	%	n	%	χ^2^	p
Poor	6	100	0	0.0	3	50.0	3	50.0	5.81	.445
Average	183	100	50	27.3	91	49.7	42	23.0		
Good	87	100	23	26.4	37	42.5	27	31.0		
Very good	28	100	8	28.6	11	39.3	9	32.1		
**Working status**	n	%	n	%	n	%	n	%	χ^2^	p
Retired	227	100	61	26.9	107	47.1	59	26.0	0.20	.907
Not retired	77	100	20	26.0	35	45.5	22	28.6		
**Perceived physical health**	n	%	n	%	n	%	n	%	χ^2^	p
Poor or average	154	100	52	33.8	75	48.7	27	17.5	15.39	< .001
Good or very good	150	100	29	19.3	67	44.7	54	36.0		
**Perceived mental health**	n	%	n	%	n	%	n	%	χ^2^	p
Poor or average	107	100	40	37.4	50	46.7	17	15.9	14.32	< .001
Good or very good	197	100	41	20.8	92	46.7	64	32.5		
	Mean	SD	Mean	SD	Mean	SD	Mean	SD	F	p
**Age**	63.46	6.85	63.49	6.49	63.03	6.99	64.19	6.98	0.74	.480
**Extraversion**	3.14	0.56	2.96	0.56	3.18	0.51	3.24	0.59	6.00	.003

Note. SD = standard deviation

### Logistic regression predicting activity level

As Table 2 depicts, Model 1 confirmed the effect of extraversion for both moderate (OR = 2.27, p = .003) and high activity levels (OR = 2.45, p = .004), when controlling for socio-demographics. Model 2 demonstrated a significant effect of extraversion for moderate activity level (OR = 2.01, p = .016) but not for high activity level (p > .05) when perceived physical health and mental health were entered, indicating potential interaction effects. Model 3 examined the main effects of extraversion, perceived physical health, perceived mental health, and their interaction effects but showed no significant results at p < .05.

**Table 2:** Multi-nominal logistic regression on activity level with extraversion and perceived health as predictors.

	Model 1	Model 2	Model 3
**Likelihood ratio *Χ***^***2***^ **(p)**	32.99 (.017)	45.09 (.003)	47.19 (.007)
**Pearson *Χ***^***2***^ **(p)**	597.83 (.316)	594.48 (.330)	594.38 (.289)
**Deviance *Χ***^***2***^ **(p)**	606.16 (.236)	596.83 (.305)	594.73 (.286)
**Cox and Snell**	.103	.138	.144
**Nagelkerke**	.117	.157	.163
**Moderate vs. low level**			
**Gender (male)**	1.10 (0.56, 2.15)	1.10 (0.56, 2.17)	1.09 (0.55, 2.16)
**Having no children**	0.72 (0.35, 1.49)	0.71 (0.34, 1.49)	0.67 (0.32, 1.41)
**Living with others**	1.70 (0.68, 4.27)	1.76 (0.69, 4.51)	1.69 (0.65, 4.38)
**Education (primary)**	0.55 (0.17, 1.71)	0.58 (0.18, 1.86)	0.56 (0.17, 1.81)
**Education (secondary)**	0.99 (0.53, 1.84)	1.04 (0.55, 1.93)	1.03 (0.55, 1.93)
**Not retired**	0.87 (0.44, 1.74)	0.84 (0.42, 1.68)	0.88 (0.43, 1.78)
**Age**	0.98 (0.94, 1.03)	0.99 (0.94, 1.04)	0.99 (0.94, 1.04)
**Household finance**	0.66 (0.42, 1.02)	0.58 (0.36, 0.93)*	0.59 (0.37, 0.94)*
**Extraversion**	2.27(1.31, 3.93)**	2.01 (1.14, 3.56)*	1.70 (0.74, 3.94)
**Perceived physical health (poor or average)**		0.78 (0.34, 1.82)	3.01 (0.24, 374.21)
**Perceived mental health (poor or average)**		0.68 (0.30, 1.52)	0.03 (0, 3.79)
**Extraversion** * **Perceived physical health (poor or average)**			0.64 (0.14, 2.94)
**Extraversion** * **Perceived physical health (good or very good)**			0
**Extraversion** * **Perceived mental health (poor or average)**			2.85 (0.58, 13.09)
**Extraversion** * **Perceived mental health (good or very good)**			0
**High vs. low level**			
**Gender (male)**	1.42 (0.68, 2.98)	1.38 (0.64, 2.97)	1.37 (0.64, 2.95)
**Having no children**	0.65 (0.28, 1.50)	0.66 (0.28, 1.55)	0.63 (0.26, 1.49)
**Living with others**	0.43 (0.17, 1.08)	0.50 (0.20, 1.29)	0.48 (0.18, 1.24)
**Education (primary)**	0.45 (0.12, 1.63)	0.48 (0.13, 1.86)	0.47 (0.12, 1.81)
**Education (secondary)**	0.75 (0.37, 1.52)	0.82 (0.40, 1.69)	0.82 (0.40, 1.69)
**Not retired**	1.12 (0.52, 2.42)	1.03 (0.47, 2.27)	1.06 (0.48, 2.35)
**Age**	1.01 (0.96, 1.07)	1.02 (0.96, 1.07)	1.02 (0.96, 1.08)
**Household finance**	0.88 (0.55, 1.43)	0.69 (0.41, 1.15)	0.70 (0.41, 1.17)
**Extraversion**	2.45 (1.33, 4.54)**	1.86 (0.98, 3.55)	1.51 (0.63, 3.62)
**Perceived physical health (poor or average)**		0.48 (0.18, 1.26)	0.67 (0.02, 178.36)
**Perceived mental health (poor or average)**		0.51 (0.19, 1.39)	0.05 (0, 20.22)
**Extraversion** * **Perceived physical health (poor or average)**			0.89 (0.15, 5.12)
**Extraversion** * **Perceived physical health (good or very good)**			0
**Extraversion** * **Perceived mental health (poor or average)**			2.21 (0.32, 15.53)
**Extraversion** * **Perceived mental health (good or very good)**			0

Note. N = 304. CI = confidence interval.

* p < 05.

** p < 01.

To reveal the potential relationship of extraversion, perceived physical health, and perceived mental health with high level of activity, model 4 was constructed to examine the prediction of extraversion for activity levels among participants with different perceived health. As shown in Table 3, extraversion was a significant predictor of either moderate (OR = 3.84, p = .014) or high (OR = 5.06, p = .032) activity level among participants with poor or average perceived mental health. This relationship did not exist for other groups.

**Table 3:** Multi-nominal logistic regression on activity level with extraversion as predictor and perceived health as grouping factor.

	Grouped by perceived mental health		Grouped by perceived physical health	
	Poor or average (n = 107)	Good or very good (n = 197)	Poor or average (n = 154)	Good or very good (n = 150)
**Likelihood ratio *Χ***^***2***^ **(p)**	44.80 (.001)	19.80 (.471)	39.12 (.006)	15.61 (.740)
**Pearson *Χ***^***2***^ **(p)**	218.55 (.076)	388.74 (.241)	310.53 (.134)	290.13 (.268)
**Deviance *Χ***^***2***^ **(p)**	172.54 (.813)	390.16 (.226)	275.73 (.626)	295.27 (.203)
**Cox and Snell**	.342	.096	.224	.099
**Nagelkerke**	.394	.109	.258	.113
**Moderate vs. low level**				
**Gender (male)**	1.17 (0.40, 3.43)	1.11 (0.44, 2.82)	1.36 (0.54, 3.42)	0.89 (0.31, 2.57)
**Having no children**	0.39 (0.11, 1.36)	0.94 (0.34, 2.57)	0.44 (0.17, 1.14)	1.60 (0.43, 6.01)
**Living with others**	1.88 (0.27, 13.18)	1.87 (0.59, 5.92)	3.30 (0.58, 18.94)	1.90 (0.51, 7.09)
**Education (primary)**	0.34 (0.06, 2.02)	0.59 (0.10, 3.39)	0.57 (0.11, 2.93)	0.59 (0.10, 3.58)
**Education (secondary)**	1.26 (0.45, 3.54)	0.95 (0.41, 2.18)	1.20 (0.53, 2.70)	0.92 (0.33, 2.59)
**Not retired**	0.34 (0.08, 1.51)	1.31 (0.53, 3.24)	0.55 (0.20, 1.54)	1.37 (0.48, 3.95)
**Age**	1.03 (0.95, 1.12)	0.94 (0.88, 1.01)	0.99 (0.93, 1.06)	0.96 (0.89, 1.04)
**Household finance**	0.65 (0.26, 1.60)	0.53 (0.30, 0.95)*	0.65 (0.32, 1.31)	0.52 (0.27, 1.00)*
**Extraversion**	3.84 (1.31, 11.24) *	1.63 (0.77, 3.44)	2.07 (0.94, 4.57)	2.14 (0.87, 5.26)
**Perceived physical health (poor or average)**	5977782.42 (5977782.42, 5977782.42)	0.66 (0.27, 1.63)	—	—
**Perceived mental health (poor or average)**	—	—	0.69 (0.29, 1.66)	0 (0,0)
**High vs. low level**				
**Gender (male)**	1.41 (0.28, 7.02)	1.33 (0.51, 3.46)	1.93 (0.56, 6.71)	1.06 (0.36, 3.09)
**Having no children**	0.71 (0.14, 3.78)	0.66 (0.22, 2.01)	0.42 (0.12, 1.56)	1.26 (0.32, 5.00)
**Living with others**	0.07 (0.01. 0.49)	0.96 (0.31, 3.02)	0.10 (0.02, 0.46)**	1.23 (0.33, 4.49)
**Education (primary)**	0.57 (0.06, 5.16)	0.40 (0.07, 2.41)	0.78 (0.11, 5.69)	0.36 (0.06, 2.30)
**Education (secondary)**	1.05 (0.23, 4.76)	0.69 (0.28, 1.69)	0.82 (0.28, 2.43)	0.67 (0.23, 1.97)
**Not retired**	4.07 (0.64, 25.68)	0.99 (0.38, 2.62)	2.42 (0.67, 8.80)	0.90 (0.29, 2.74)
**Age**	1.08 (0.96, 1.21)	0.99 (0.92, 1.06)	1.01 (0.93, 1.10)	1.02 (0.94, 1.10)
**Household finance**	0.47 (0.10, 2.28)	0.73 (0.40, 1.32)	0.75 (0.29, 1.93)	0.64 (0.33, 1.25)
**Extraversion**	5.06 (1.15, 22.20) *	1.45 (0.66, 3.17)	2.73 (0.91, 8.18)	1.78 (0.72, 4.45)
**Perceived physical health (poor or average)**	1.58 (0.02, 119.96)	0.44 (0.16, 1.20)	—	—
**Perceived mental health (poor or average)**	—	—	0.48 (0.15, 1.54)	0.91 (0.04, 20.12)

Note. N = 304. CI = confidence interval.

* p < 05.

** p < 01.

## Discussion

This study echoes previous research findings that active participation in a wide range of activities is increasingly recognized as a contributor to well-being in aging adults [2, 28]. It adds knowledge to understanding individual determinants of activity levels among aging adults, specifically extravert personality and its interaction with perceived physical and mental health. Similar to previous research studies [7, 13, 26], this study supports the positive association between extravert personality and activity participation. However, this positive association is not simply linear but affected by perceived health status, as extravert personality might increase the likelihood of moderate but not high activity levels when controlling for health status. Extraversion increases the odds of both moderate and high activity levels only for participants with poor or average perceived mental health. This signifies the importance of motivations provided by extravert personality for aging adults with impaired mental health to remain highly active.

In the past, studies on activity participation focused mainly on how behavioral change (to be more active) could be achieved. This study shows that a focus on personality factors could be equally important. High extraverts are found to prefer highly stimulating social situations (e.g. social gatherings, sports, leisure) [50]. This has been ascribed to a stronger behavioral activation system in the brain compared to low extraverts, which stimulates intention or motivation for activity participation [51].

The positive associations between perceived physical health and activity level reported in this study confirm the barrier effect postulated in the model of constraints [21]. Physical dysfunction negatively affects aging adults’ capacity and intention to participate in activities. As previous studies have illustrated, frailty reduces the vigor essential for activity engagement [27], physical disability limits mobility and communication [7, 52], and pain or other symptoms cause reduced interest in activities [39]. Worry about public rejection due to one’s physical condition is also a probable interpersonal barrier to activity participation [42].

The findings related to perceived mental health are complex. Its positive association with activity level provides significant support to the barrier (rather than compensation) proposition. Mental health impairments can harm aging adults’ sense of surroundings and inner behavioral motivation mechanisms to enter social contexts for activity participation [41]. As interpersonal barriers, limitations in mental strength, mood, and cognition can dramatically reduce a person’s capacity and intention to participate in activities [41]. Social stigma and discrimination associated with mental illness can also represent interpersonal barriers and isolate people from activities [53]. However, older adults with impaired mental health could also achieve high activity levels given extravert personality. This could be explained by the compensation proposition [32]. Extravert aging people may tend to more actively participate in social activities in order to compensate for the negative influence of mental illness or disorder on their social life.

These findings provide important empirical evidence to inform policy making and service provision to support active aging. Both prevention and intervention approaches are needed to increase activity participation among aging adults. Concerning the impact of health on activity participation, health enhancement measures should be provided or improved, including but not limited to health promotion, nutrition, immunization, and preventive rehabilitation. To increase activity participation among aging people with impaired health, interventions such as medication, surgery, rehabilitation, and health care interventions could contribute to functional recovery or capacity improvement. Aging adults, particularly those with health impairments, require more attention from policy makers and service providers to reduce interpersonal and structural barriers to participation, through the promotion of age-friendly physical environments (e.g. universal design, environment accommodation) and social atmospheres (e.g. public education for anti-discrimination and accessible services) [33]. The findings on the effect of extravert personality point to directions for the development and implementation of inclusive and stimulating activities or programs to increase aging people’s intention and motivation to participate in activities, particularly for those who do not exhibit extroversion characteristics. Tailored programs that focus on providing stronger stimulants or motivators for soliciting activity participation by aging people with introversion traits could be useful. For these aging people, use of interpersonal and social connections could be helpful to bring them out of their comfort zone [7]. The use of peer support or peer mentors [14] to motivate their interest in activity could be further examined.

The major limitation of this study lies in its cross-sectional approach. Extraversion, perceived health, and activity participation may coexist in a recursive loop. More specifically, activities (e.g. sports, leisure) have physical and social consequences (e.g. physiology, self-esteem, expectations) for personality functioning [23, 50]. However, it is beyond the capacity of this study to determine the causal effects between these variables. Additionally, the external validity of the data was limited as the selected sample only included 50% of registrants in programs provided by an institute of active aging, which may encourage activity participation to some extent, and as such may not represent aging people more generally, especially those who are homebound or experiencing social isolation. Therefore, the representation of the resulting sample has to be interpreted with caution. Aside from extraversion, other personality dimensions such as neuroticism and conscientiousness could influence the activity level of older adults but would be left to future research due to the limited capacity of this study. Furthermore, the robustness of the measure of extraversion could be reduced through telephone interviews. The single question measure of overall activity level could be limited in its validity. Finally, the accuracy of the findings might be affected by the retrospective nature of the data. Future research endeavors could be strengthened by conducting longitudinal research involving larger and more representative samples.

## Supporting information

S1 File (SAV)
