# Correction: Extraversion personality, perceived health and activity participation among community-dwelling aging adults in Hong Kong

**DOI:** 10.1371/journal.pone.0227896

**Published:** 2020-01-13

**Authors:** Daniel W. L. Lai, Nan Qin

The first author, Daniel W. L. Lai, is incorrectly noted as the corresponding author. The correct corresponding author is Nan Qin. Dr. Qin’s email address is: nanqin@gdufe.edu.cn.

## References

[1] Lai DWL, Qin N (2018) Extraversion personality, perceived health and activity participation among community-dwelling aging adults in Hong Kong. PLoS ONE 13(12): e0209154 10.1371/journal.pone.0209154 30540853 PMC6291235

